# Optimized CyberKnife Lung Treatment: Effect of Fractionated Tracking Volume Change on Tracking Results

**DOI:** 10.1155/2020/9298263

**Published:** 2020-01-11

**Authors:** Guo-quan Li, Ye Wang, Meng-jun Qiu, Jing Yang, Zhen-jun Peng, Sheng Zhang, Xiefan Fang, Sheng-li Yang

**Affiliations:** ^1^Cancer Center, Union Hospital, Tongji Medical College, Huazhong University of Science and Technology, Wuhan 430022, China; ^2^Division of Gastroenterology, Liyuan Hospital, Tongji Medical College, Huazhong University of Science and Technology, Wuhan 430077, China; ^3^Charles River Laboratories, Inc., 6995 Longley Lane, Reno, NV 89511, USA

## Abstract

**Objectives:**

To explore the impact of volume change in the fractionated tracking of stereotactic radiotherapy on the results of synchronous, respiratory tracking algorithm using CyberKnife.

**Methods:**

A total of 38 lung tumor patients receiving stereotactic radiotherapy at our center from March 2018 to October 2019 were counted. Photoshop CS4 image processing software was used to obtain the pixels and the average value of brightness of the tracking volume in the image and calculate the grayscale within the contour of the tracking volume on the real-time X-ray image. At the same time, parameters of the synchronous respiratory tracking algorithm of the fractional CyberKnife were extracted for comparison between the volume of image-guided image tracking and the number of fractions during stereotactic radiotherapy. We also analyzed the relationship between fraction tumor location and characteristics and the calculated results of synchronous respiratory tracking by CyberKnife.

**Results:**

There were no significant differences between the first four fractions (*p* > 0.05) for left lung lesions and no significant differences between the first five fractions for right lung lesions (*p* ≥ 0.05). For peripheral lung cancer, longer fractional treatment led to greater variation in grayscale (G-A: >4 fractions *p* < 0.05), while for central lung cancer, longer fractional treatment led to greater variation in parameters of the synchronous respiratory tracking algorithm (Uncertainty A and Uncertainty B: >4 fractions *p* < 0.05). There was a significant correlation between radiotherapy-graded tumor density and relevant parameters, and the correlation was strong (>0.7, *p* < 0.05).

**Conclusion:**

With the increase of treatment fractions, the gray value in the patient tracking volume decreased. Patients of >4 fractions were advised to reevaluate with simulated CT and replan. For tumors with small diameter and low density, the imaging changes of volume should be closely followed during treatment. For left lung and central lung cancer, carefully select the synchronous tracking treatment with 2-view.

## 1. Introduction

In recent years, advances in image guidance and focused irradiation technology have brought radiotherapy into an era of precision and efficiency [1]. With the advent of CyberKnife, a new radiosurgery system in 2001 [2], stereotactic radiotherapy has become a research hotspot. CyberKnife helped solve the physical, technological problems in radiotherapy [3], and its submillimeter imaging guidance accuracy and synchronous respiratory tracking technology provide an alternative for clinical stereotactic radiotherapy of lung tumors [4–7]. A treatment course can be completed using 3-5 fractions if a single high dose is administered [8]; however, practically, our center considers a patient's tolerance clinically and usually performs 5-9 fractions or radiotherapy every other day. With an increase in the number of fractions, the target tracking volume of some patients' changes on the interactive X-ray guided images, impacting the results calculated by synchronous respiratory tracking in the clinical treatment of lung tumors in a certain way. This impact results in the poor quality of respiratory models during treatment, frequent reconstruction of models, and affects the efficiency of treatment.

Furthermore, for these cases, one cannot judge whether the tracking volume would change under the current fraction before treatment and whether it would affect the results of the algorithm calculation. Therefore, this study retrospectively analyzed cases that had completed the simultaneous respiratory tracking therapy with CyberKnife and explored the changes of tracking volume in different fractionated treatments and the impact on the calculation results of CyberKnife tracking algorithm to provide some reference for clinicians to choose the appropriate fractionation method and the timing of intervention modification plans.

## 2. Materials and Methods

### 2.1. Clinical Materials

38 lung cancer patients undergoing stereotactic radiotherapy at our center from March 2018 to October 2019 were selected. 25 of the patients were male, and 13 were female. The ages of the patients were between 41 and 76 (average age of 58.5). Radiotherapy was provided 5-7 times, as shown in Table 1. Stereotactic radiotherapy using the CyberKnife synchronous respiratory tracking technology was performed one time per day from Monday to Friday (Saturdays and Sundays were rest days). All patients were treated on the CyberKnife VSI system using 2-view XLTS.

Patient characteristics are presented in Table 1, categorized by anatomical location (upper, middle, and lower lobe) and degree of centrality (central vs. peripheral). Central lesions are located within or touching the zone of the proximal bronchial tree [9], defined as a volume 2 cm in all directions around the proximal bronchial tree, while lesions outside this area are defined as peripheral. Tumor volume, density, and tumor size are also reported in Table 1.

### 2.2. Equipment Information

The following are the equipment information: American Accuray Company CyberKnife VSI (with standard treatment couch), treatment plan system MultiPlan 5.2.1, and treatment execution system CyberKnife 10.5.

### 2.3. CT Simulation, Tracking Volume Definition, and Treatment Plan

All patients performed vacuum pad fixation. The section thickness of 1.5 mm was used for CT scan under large aperture CT simulator. The range is 15 cm above and below the target area of the tumor, including all the organs at risk around the target area. Transfer CT images to MultiPlan v. 5.2.1 (Accuray Inc.) treatment planning system (TPS). The clinical target area was mapped from the lung cancer atlas by the same experienced radiation oncologist. The tracking volume for the 2-view synchrony tracking is depicted by the same experienced medical physicist. The tracking volume is only used as a reference object for Synchrony Systems during treatment. Different from the clinical target area of tumor, it should be composed of solid structures with high density of tumor lesions on CT images. According to the training of CyberKnife manufacturers, the tracking volume should be depicted on the CT image close to the medial edge of the lung lesion entity and should not include the foggy and burr part.

According to training suggestions of CyberKnife manufacturers, CyberKnife simulation module was used for tumor visualization simulation of all patients before XLT treatment. All patients were clearly visible on A and B images, and the tumor had a minimum diameter of 1.5 cm in any direction.

The CyberKnife planning system adopts the reverse optimization algorithm to design and optimize the radiotherapy plan, so that 95% volume of PTV of each plan can obtain the clinical prescription dose value. The limited dose was administered with reference to report the Radiation Therapy Oncology Group (RTOG) 023. The relationship between fractions and image parameters and the relationship between fractions and synchronous respiratory tracking algorithm parameters were both analyzed.

Based on this study, the location and characteristics of the tumor were predicted to be related to the image parameters and tracking algorithm parameters of the patient. The correlation between the density, volume, and location of the tumor (left/right lung or peripheral/central type) before treatment and the image parameters and tracking algorithm parameters was also analyzed.

### 2.4. Image Parameter Setting and Calculation

**Figure 1:**
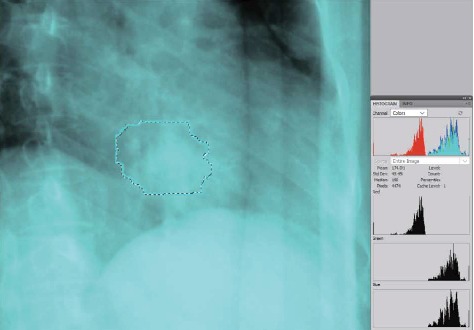
The image processing software obtains the pixel and average brightness values of the tracking volume in the image. Note: the solid blue contour in the figure is the projection of tumor tracking volume on real-time X-ray images. The black dotted outline is the area where the graph processing software calculates the density and mean brightness.

For all patients, the parameters of X-ray imaging in each fraction was the default value of treatment execution system (120 kV, 100 mA, 100 ms), and the image interval was set as 90 s. Will breathe the halfway point (the midpoint between the previous crest and the next trough) of the phase is set to the starting position of the synchrony model (according to the CyberKnife manufacturer recommendations, the first starting point should guarantee three offset 0), and then use the F12 key record camera A and camera B image. The image format obtained is JPG, and the resolution of the image is 3840 × 1200. Use Adobe Photoshop CS4 graphics processing software to obtain the average pixel and brightness of the tracking volume in the image (Figure 1).

#### 2.4.1. Pixels Per Inch (PPI)

Unit of image resolution that represents the number of pixels per inch. The higher the PPI, the more detail the image shows. The pixel density is obtained by the following formula: [10]

(1) $$\begin{array}{c} \text{PPI}=\frac{\sqrt{N_{l}^{2}+N_{l}^{2}}}{l\times w}, \end{array}$$

where PPI represents the pixel density; *N*_*l*_ is the number of length pixels; *N*_*w*_ is the number of width pixels; *l* × *w* is the screen size.

#### 2.4.2. Grayscale

Because the differences in image resolution between observers were based on the difference in image grayscale, therefore, the grayscale changes in the patient's volume during treatment can describe the tumor changes. The following formula expressed the grayscale.

(2) $$\begin{array}{c} g=f\times \bar{I}, \end{array}$$

where *g* represents gray, *f* represents the pixels, and $\bar{I}$ represents the average brightness.

### 2.5. Synchronous Respiratory Tracking Algorithm Parameters by CyberKnife

CyberKnife Synchrony Systems implementing breath tracking management technology initially adopts the least mean square (LMS) linear algorithm [11]. Then, it is improved to match the breathing signal that is most similar to the current breathing cycle in the historical breathing data as a reference to predict breathing movement, which is called pattern match algorithm [12]. Synchrony's latest hybrid algorithm [13–15] using least squares method and pattern matching combined with fuzzy logic to predict tumor respiration. At present, Synchrony Systems has been widely used in clinical practice, with good real-time tracking ability.

#### 2.5.1. *x*-Axis Target Pairing Tolerance (dxAB)

This parameter represented the relative distance along the head-to-foot direction of the positioning center between two projections (camera A and camera B for X-ray imaging system); the difference was called dxAB. Head and foot directions were used because the axis was common to both projections. The synchronous respiratory tracking algorithm obtains dxAB values for each image.

#### 2.5.2. Uncertainty%

Uncertainty parameter was defined as the dissimilarity between the total gray distribution of the tracking area locked on live image and the total gray distribution of the tracking area defined by the dosimetrist on the DRR image. Uncertainty% was expressed using the following formula:

(3) $$\begin{array}{c} \text{Uncertainty}\%=\left(1-\frac{N_{\text{DRR}}}{N_{\text{Live}}}\right)\times 100\%, \end{array}$$

where *N*_DRR_ is the number of pixels of the DRR image reconstructed for locating CT, and *N*_Live_ is the number of pixels of a real-time X-ray image.

### 2.6. Statistical Processing

The Kolmogorov-Smirnov (KS) test results of data samples all obeyed normal distribution (*p* > 0.05). After the homogeneity test of variance, one-way ANOVA using the SPSS 22.0 software was used to analyze the interfraction differences between image parameters and algorithm parameters. Pearson correlation was used to perform a supplementary analysis of the correlation strength between factions and image parameters/algorithm parameters. Data is represented as the mean ± SD. The difference was considered statistically significant if *p* < 0.05.

## 3. Results

### 3.1. The Impact of Fractionated Treatment on Image Parameters

**Figure 2:**
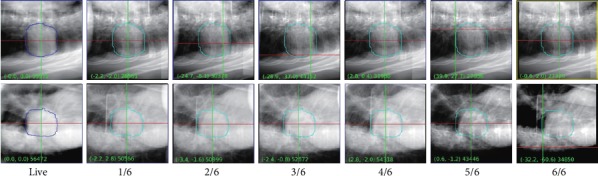
Comparing pixel density differences among real-time X-ray images of a 6-fraction case. Note: image guidance of a 6-Fractions patient. In the figure, live is the DRR image reconstructed from the CT localization image, and each sequence number is arranged as the real-time X-ray image taken in daily intervals.

Table 2 contains summaries of the image parameters for 1 to 7 therapies. The grayscale of the tracking volume shows a monotonous decline on the two images of A and B.  Figure 2 shows the daily real-time X-ray image comparison of one patient. With the increase of fraction, the shadow of the tumor in the contour of the tracking volume gradually faded.

### 3.2. Effect of Tumor Location on Image Parameters

Table 3 shows the difference in pixel density percentages of image-guided images between 2-7 and the first radiotherapy. With increasing fractional treatment, the percentage difference of the pixel density between the A and B images of the left lung lesions seemed to be increasing, but the difference between the first four fractions was not significant (*p* > 0.05); only the percentage difference of the pixel density in the B image of the right lung lesions was increasing, with the difference between the first five fractions not significant (*p* ≥ 0.05).  Table 4 shows the image guidance images of the first radiotherapy as the standard reference, and all the image parameters are increasing with the fractional changes. However, *p* < 0.05 for Uncertainty A of the left lung after 6 fractions.

For peripheral lung cancer, longer subdivision leads to greater variation in gray level (G-A: >4 fractions, *p* < 0.05). However, for central lung cancer, the longer the treatment time, the greater the variation of tracking algorithm parameters (Uncertainty B and Uncertainty < 0.05) (Table 5).

### 3.3. Effect of Tumor Characteristics on Image Parameters and Algorithm Parameters

Table 6 shows Pearson correlation analysis results including tumor density, volume, size, and segmentation with image parameters. There was a significant and strong correlation between tumor density and related parameters (>0.7, *p* < 0.05). The tumor size was only significantly correlated with the parameters of the algorithm (Uncertainty B, Uncertainty A, and dxAB) (*p* < 0.05). However, there was no significant correlation between tumor volume except dxAB and related parameters (dxAB, *p* < 0.05).

The change in the image-guided pixel density was more apparent due to the change in fractionation, but the changes in the parameters of the synchronous respiratory tracking algorithm were not as evident as the visual percentage change in pixel density. Table 6 lists the correlation coefficients of each correlation parameter and the percentages of pixel density with fractions. The results show that the correlation coefficient of the percentage of the pixel density of the left lung lesion B image was -0.757, while the correlation coefficient of the percentage of the pixel density of the right lung lesion A image was -0.507. The correlation coefficient of the left lung image dXAB was 0.661 and that of the right lung image dXAB was 0.169. The correlation coefficient of the left lung Uncertainty B was 0.465 and that of the right lung Uncertainty A was 0.348.

## 4. Discussion

As far as we know, many researchers [16–20] have proven that the geometric changes of target areas in patients can be triggered by weight loss, organ deformation, and tumor contraction, among other reasons during the entire fractionated radiotherapy cycle routine. Furthermore, it has been proven that rescheduling after a certain fraction for some patients receiving intensity-modulated radiation therapy (IMRT) and volumetric-modulated arc therapy (VMAT) can achieve significant benefits. During conventionally fractionated radiotherapy, some researchers commonly use dose calculations based on CBCT to decide if they need to reschedule. Fotina et al. [21] studied the density of all structures of interest on manual “overriding” CBCT images based on conventionally fractionated radiotherapy. Because 2-5 fractionated stereotactic radiotherapy is considered to have a short treatment cycle and few target area changes, it is unlikely that it will attract attention. However, in actual clinical treatment processes, radiotherapists may prescribe 5-8 times of radiotherapy fractions based on the actual situation of patients. The change in tracking volume caused by small tumor changes during a radiotherapy cycle leads to a corresponding change in parameter outputs by the synchronous tracking algorithm. Per the results of this research, the parameters of the synchronous respiratory tracking algorithm increased with an increase in the number of fractions, and a few cases appeared to exceed the system's preset maximum threshold set by the radio knife system by default. So, in the end, the plan needed to be reevaluated in collaboration with a competent physician to complete the course of radiotherapy.

Accuray Company's CyberKnife VSI system uses a unique synchronous respiratory tracking technology to achieve accurate stereotactic thoracic radiotherapy. For accuracy sake, although the system has corresponding tracking algorithms to calculate the location of tumors, it is very important that therapists confirm the tracking position of the tumors visually. That notwithstanding, tracking errors caused by uncertainty may still vary between 2 and 6 mm [22]. Therefore, the rise in uncertainty could lead to a greater deviation. This present study shows that there was no significant difference in the grayscale of image guidance parameters between, at least, the first four fractionated tracking volumes, and statistical significance only emerged after the first four fractions.

Although the resulting differences in output parameters of synchronous respiratory tracking were not synchronous, Pearson correlation analysis showed that the output parameters of synchronous respiratory tracking correlated positively with fractions and negatively with image parameters. These findings indicate that the increase in the number of fractions may also cause a change in output parameters of synchronous respiratory tracking. The change in image parameters would, at the very least, affect the visual judgment of therapists. Therefore, it is suggested that the treatment plan for the pulmonary SBRT fraction exceeding 4 fractions should take into consideration the change in grayscale of the tracking volume. The fourth fraction should be carefully monitored when the treatment cycle takes place over a weekend to revise the tracking volume to implement tracking therapy better.

**Figure 3:**
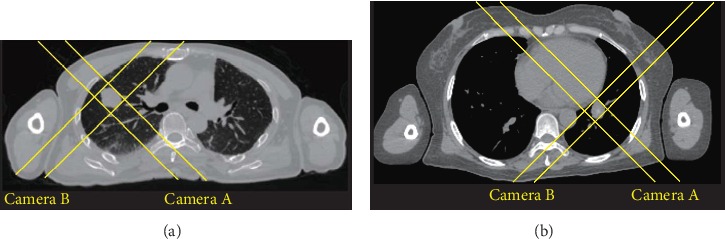
Schematic diagram of cross-field image guidance for peripheral lung cancer and central lung cancer. Note: (a) transverse image of a patient with peripheral lung cancer and (b) transverse image of a patient with central lung cancer.

We also attempted to investigate the differences in tumor location (central/peripheral and left/right lung) in CyberKnife image-guided image parameters, as well as synchrony tracking parameters. According to the results obtained, CyberKnife image-guided B images for the left lung lesions correlated strongly with fraction changes and with the Uncertainty B% of synchronous respiratory tracking output parameters. However, the outcomes for the right lung lesions were contrary to those of the left. Therefore, for left lung lesions, the tracking parameters results may be better when using the B image for treatment if there is need to implement the 1-view tracking mode, whereas, for right lung lesions, the image results using the A image would be a better fit. For peripheral lung cancer, longer segmentation results in larger gray variation, while synchrony tracking output parameters do not change much. On the contrary, for central lung cancer, the tracking algorithm parameters changed more with longer treatment. The reason of this phenomenon may be related to the unique 45° orthogonal field of view (Camera A and Camera B) of CyberKnife. As shown in Figure 3, the tracking volume of central lung cancer is more susceptible to the background of opaque structures such as the spine and heart, while peripheral lung cancer is less affected [23].

Pathak et al. [24], using KVCT obtained before each fractional treatment, analyzed the changes that might occur during SBRT in 22 lung cancer patients. Their results showed that total tumor volume changed significantly during SBRT. 45.5% tumor volume decreased relative to the first subdivision. In our study, patients who could not be modeled at all (the tracking algorithm could not correctly identify the tumor or the therapist could not visually determine that the tracking system was tracking incorrectly) due to the large changes of the grayscale in the tracking volume in the image-guided image during treatment were not included in the study. This usually occurs after the patient has received fourth fraction of treatment. If this happens, radiologists and physicists need to reacquire the CT simulation and change the treatment plan. The apparent change in tracking volume may be due to the synergistic effect of radiotherapy combined with chemotherapy or other treatments, leading to rapid tumor regression. It is also possible that the patient developed pleural effusion during treatment [25].

Also, the difference between observers is a familiar problem in medical practice. Gandevia and Stradling first reported this problem in the fifties of the twentieth century [26]. In recent years, there have been many studies on the differences between observers and computers in the field of radiotherapy [27, 28]. Our study did not analyze the parameter differences of synchronous respiratory tracking output resulting from different observers' perception of tracking volume boundaries. This nonincidence, however, posed no effect on counting the parameter changes of the synchronous respiratory tracking output caused by the change in pixel density in the tracking volume region. The correlation coefficient between fraction and image parameters was found to be stronger than that between fraction and synchronous respiratory tracking output parameters. The possible reason is that the synchronous respiratory tracking algorithm calculates other correlation factors included in the image beside the pixel density difference. Our study did not examine the differences in tracking output parameters caused by other factors (such as pathological type of tumor, exposure conditions, patient positions, among others) in image guidance. Hence, our conclusion may only serve as a random result of the left and right lung lesions, with further studies needed to confirm our findings.

This study, like most first time studies, has its limitations. First of all, the sample size for the research was small, and a significant number of clinical trials would be required to verify our results. Secondly, relevant authoritative scales for the more accurate assessment of image differences caused by tracking volume changes are in shortage. Finally, this is a single-agency study, and different versions of the system may have different tracking algorithms. Higher versions of the system may compensate for the image differences among fractions, making it more accurate in tracking target treatment, meaning such results may be slightly different from ours.

## Figures and Tables

**Table 1:** Clinical characteristics of 38 patients.

Characteristics	
Gender	
Male	25
Female	13
Anatomical location	
Upper lobe	15
Middle lobe	13
Lower lobe	10
Tumor location	
Peripheral	17
Central	21
Treat side	
Left	18
Right	20
Fraction	
5	13
6	11
7	14
Tumor density: mean ± SD/median (g/cm^3^)	0.86 ± 0.08/0.83
Tumor volume: mean ± SD/median (cm^3^)	13.4 ± 7.8/7.7
Tumor size (the shortest lengths in three dimensions): mean (cm)	3.4∗2.5∗2.3

**Table 2:** Statistics of parameters related to the tracking therapy ($\bar{x}\pm s$).

Fraction	G-B (×10^4^)	G-A (×10^4^)	dxAB	Uncertainty B	Uncertainty A
1	86.3 ± 7.6	75.1 ± 13.1	0.64 ± 0.75	9.4 ± 6.9	8.5 ± 3.4
2	85.7 ± 9.2	74.7 ± 15.5	0.72 ± 0.86	10.0 ± 7.3	9.7 ± 5.9
3	84.0 ± 8.9	72.3 ± 11.6	0.80 ± 0.93	13.0 ± 9.4	12.9 ± 8.0
4	80.5 ± 11.4	62.6 ± 17.9	1.10 ± 1.23	14.1 ± 10.9	13.7 ± 6.7
5	75.3 ± 14.7	55.3 ± 18.3	1.83 ± 1.27	16.8 ± 11.7	16.9 ± 7.9
6	67.1 ± 17.2	46.7 ± 15.9	1.35 ± 0.98	17.2 ± 4.6	12.8 ± 3.7
7	66.2 ± 8.4	44.1 ± 13.5	1.47 ± 0.86	18.9 ± 12.3	14.5 ± 8.1

Note: G: grayscale.

**Table 3:** Comparing grayscale differences of the tracking volume between the first and >1 fractionated radiotherapy (least significant difference: LSD).

(i) fraction	(j) fraction	Left lung		Right lung	
		G-B	G-A	G-B	G-A
1	2	0.928	0.958	0.948	0.781
	3	0.502	0.843	0.792	0.496
	4	0.054	0.496	0.361	0.119
	5	0.001	0.141	0.077	0.005
	6	0.0	0.013	0.022	0.0
	7	0.0	0.035	0.118	0.0

Note: G: grayscale.

**Table 4:** Comparing parameters of the synchronous respiratory tracking algorithm between the first time and >1 fractionated radiotherapy (least significant difference: LSD method).

(i) fraction	(j) fraction	Left lung			Right lung		
		Uncertainty B	Uncertainty A	dxAB	Uncertainty B	Uncertainty A	dxAB
1	2	0.898	0.933	0.901	0.883	0.693	0.809
3		0.723	0.610	0.708	0.406	0.146	0.653
4		0.501	0.792	0.248	0.279	0.095	0.029
5		0.149	0.171	0.022	0.088	0.008	0.002
6		0.013	0.020	0.001	0.160	0.274	0.214
7		0.072	0.774	0.000	0.346	0.311	0.262

Note: the chart shows the single-factor multiple comparison results of the tracking algorithm parameters of the first fractional radiotherapy with >1. The table shows the image guidance images of the first radiotherapy as the standard reference, and all the image parameters are increasing with the fractional changes. However, *p* < 0.05 is for Uncertainty A of the left lung after 6 fractions.

**Table 5:** Peripheral/central lung cancer was compared for the first fraction with >1 fractional radiotherapy image parameters/tracking algorithm parameters (least significant difference: LSD method).

Tumor location	Fraction	G-B	G-A	Uncertainty B	Uncertainty A	dxAB
Peripheral	2	0.941	0.911	0.967	0.876	0.908
	3	0.700	0.714	0.583	0.319	0.785
	4	0.686	0.547	0.532	0.058	0.022
	5	0.132	0.030	0.107	0.018	0.001
	6	0.043	0.001	0.110	0.246	0.244
	7	0.000	0.000	0.215	0.140	0.079

Central	2	0.970	0.875	0.853	0.795	0.775
	3	0.761	0.673	0.347	0.310	0.643
	4	0.355	0.194	0.079	0.053	0.149
	5	0.081	0.112	0.004	0.001	0.200
	6	0.073	0.090	0.000	0.000	0.019
	7	0.054	0.005	0.000	0.000	0.001

Note: G: grayscale.

**Table 6:** The correlation coefficients between tumor characteristics and related parameters.

	G-B	G-A	dxAB	Uncertainty B	Uncertainty A
Fractions	-0.873^∗∗^	-0.785^∗^	0.616^∗^	0.756^∗∗^	0.713^∗^
Tumor density	0.937^∗∗^	-0.807^∗∗^	0.778^∗^	-0.826^∗∗^	-0.676^∗^
Tumor volume	0.314	0.374	0.498^∗^	-0.137	0.240
Tumor size	0.402	0.456	-0.519^∗^	-0.633^∗^	-0.507^∗^

Note: G: grayscale. ^∗∗^The correlation was significant at 0.01 level. ^∗^The correlation was significant at 0.05 level.

## Data Availability

The data used to support the findings of this study are available from the corresponding author upon request.
